# The Emerging Fish Pathogen *Flavobacterium spartansii* Isolated from Chinook Salmon: Comparative Genome Analysis and Molecular Manipulation

**DOI:** 10.3389/fmicb.2017.02339

**Published:** 2017-11-30

**Authors:** Shicheng Chen, Jochen Blom, Thomas P. Loch, Mohamed Faisal, Edward D. Walker

**Affiliations:** ^1^Department of Microbiology and Molecular Genetics, Michigan State University, East Lansing, MI, United States; ^2^Bioinformatics and Systems Biology, Justus-Liebig-University, Giessen, Germany; ^3^Department of Pathobiology and Diagnostic Investigation, College of Veterinary Medicine, Michigan State University, East Lansing, MI, United States; ^4^Department of Fisheries and Wildlife, College of Agriculture and Natural Resources, Michigan State University, East Lansing, MI, United States

**Keywords:** *Flavobacterium spartansii*, genome analysis, virulence factors, mutation

## Abstract

*Flavobacterium spartansii* strain T16^T^ was isolated from a disease outbreak in hatchery-reared Chinook salmon (*Oncorhynchus tshawytscha*) fingerlings. To gain insight into its genomic content, structure and virulence pathogenesis factors, comparative genome analyses were performed using genomes from environmental and virulent *Flavobacterium* strains. *F. spartansii* shared low average nucleotide identity (ANI) to well-known fish-pathogenic flavobacteria (e.g., *F. columnare, F. psychrophilum*, and *F. branchiophilum*), indicating that it is a new and emerging fish pathogen. The genome in T16^T^ had a length of 5,359,952 bp, a GC-content 35.7%, and 4,422 predicted protein-coding sequences. *Flavobacterium* core genome analysis showed that the number of shared genes decreased with the addition of input genomes and converged at 1182 genes. At least 8 genomic islands and 5 prophages were predicted in T16^T^. At least 133 virulence factors associated with virulence in pathogenic bacteria were highly conserved in *F. spartansii* T16^T^. Furthermore, genes linked to virulence in other bacterial species (e.g., those encoding for a type IX secretion system, collagenase and hemolysin) were found in the genome of *F. spartansii* T16^T^ and were conserved in most of the analyzed pathogenic *Flavobacterium*. *F. spartansii* was resistant to ampicillin and penicillin, consistent with the presence of multiple genes encoding diverse lactamases and the penicillin-binding protein in the genome. To allow for future investigations into *F. spartansii* virulence *in vivo*, a transposon-based random mutagenesis strategy was attempted in *F. spartansii* T16^T^ using pHimarEm1. Four putative gliding motility deficient mutants were obtained and the insertion sites of pHimarEm1 in the genome of these mutants were characterized. In total, study results clarify some of the mechanisms by which emerging flavobacterial fish pathogens may cause disease and also provide direly needed tools to investigate their pathogenesis.

## Introduction

Among the fish diseases that result in significant losses in farmed and wild fish populations around the world, those caused by flavobacteria (Family Flavobacteriaceae, Phylum Bacteroidetes) occupy a central position (Starliper and Schill, 2011). Although the majority of published reports on flavobacterial disease outbreaks in fish, along with the research aimed at combatting them, has focused on three *Flavobacterium* spp. (*Flavobacterium psychrophilum, F. columnare*, and *F. branchiophilum*) (Starliper and Schill, 2011; Loch and Faisal, 2017), multiple novel *Flavobacterium* spp. have recently emerged in association with fish disease outbreaks (reviewed in Loch and Faisal, 2015). Interestingly, some of these novel flavobacteria not only cause systemic disease outbreaks in fish, but also generate disease signs that mimic their better-known fish-pathogenic counterparts (Loch and Faisal, 2016), thereby complicating disease diagnosis and treatment. Unfortunately, very little is known about the disease ecology, transmission, and pathogenesis of emergent fish-associated flavobacteria.

Despite decades of research, effective means for the prevention and control of flavobacterial diseases have yet to be realized. However, with the advancement and ready availability of complete genome sequencing technologies for microbes, there have been significant efforts to elucidate the molecular mechanisms behind flavobacterial pathogenicity and thereby guide vaccine development through genome sequencing of pathogenic and environmental flavobacteria alike. Indeed, complete genomes are available for a handful of *Flavobacterium* spp. (McBride et al., 2009; Barbier et al., 2012; Kumru et al., 2017) and additional genome sequence data are now available for multiple strains of the fish pathogens *F. columnare* (Bartelme et al., 2016; Zhang et al., 2016), *F. psychrophilum* (Wiens et al., 2014; Wu et al., 2015), and *F. branchiophilum* (Touchon et al., 2011). These studies have revealed some of the processes by which flavobacterial fish pathogens are believed to colonize, attack, and survive within fish hosts (Duchaud et al., 2007; Castillo et al., 2016), as well as how some of the molecular mechanisms vary amongst strains (Wiens et al., 2014; Wu et al., 2015; Bartelme et al., 2016; Zhang et al., 2016).

Recently, we described the novel species *Flavobacterium spartansii*, which was originally isolated from a disease outbreak in hatchery-reared Chinook salmon (*Oncorhynchus tshawytscha*) fingerlings and from systemically-infected, feral, spawning Chinook salmon (Loch and Faisal, 2014a). Subsequent studies investigating the pathogenicity of two *F. spartansii* strains (T16^T^ and S12) under laboratory conditions showed that they were capable of inducing morbidity and mortality in intraperitoneally injected Great Lakes Chinook salmon, although the estimated median lethal dose was relatively high and varied by strain (Loch and Faisal, 2016). Nevertheless, the induced gross and microscopic changes were severe at times, which may suggest that *F. spartansii* is a facultative salmonid pathogen. Therefore, a detailed examination of its possible pathogenicity is warranted. Through functional and comparative genomic analyses, the following study aimed to: (a) better understand the mechanisms of virulence for *F. spartansii* and investigate how its genetic repertoire compares with the better-known fish-pathogenic flavobacteria through the use of complete genome sequencing; (b) examine the antimicrobial resistance capabilities in *F. spartansii*; (c) further characterize the evolution of virulence among *Flavobacterium* species; and (d) develop additional molecular tools for genetic manipulation of flavobacteria.

## Materials and methods

### Culture conditions

*Flavobacterium spartansii* (T16^T^) was grown aerobically in CYE broth. *Escherichia coli* DH5α was used for cloning and plasmid propagation. *E. coli* S17 (λ *pir*) was used for conjugation. *E. coli* EC100D *pir*^+^ was used to recover transposons from *F. spartansii*. *E. coli* strains were routinely grown in Luria-Bertani (LB) broth. Liquid cultures were incubated with shaking (ca. 200 rpm) at either 28°C (*F. spartansii*) or 37°C (*E. coli*). For solid LB medium, Bacto agar (Difco, Detroit, MI) was added at a final concentration of 20 g/liter with kanamycin (50 μg/ml) or ampicillin (100 μg/ml) added for plasmid selection in *E. coli* or erythromycin (Em) added (100 μg/ml) for transposon or plasmid selection in *F. spartansii*. Demonstration of motility was done by culturing *F. spartansii* cells on PY2 agar medium at 28°C.

### Molecular manipulation

Isolation and purification of bacterial genomic DNA was performed with the Wizard Genomic DNA Purification Kit (Promega, CA, USA) according to the manufacturer's instruction. The purity, quality and quantity of purified genomic DNA were assessed using a NanoDrop 2000 UV-Vis spectrophotometer (Thermo Scientific, MA, USA) and a Qubit 2.0 fluorometer (Life Technologies, MA, USA), respectively.

### Random transposon mutagenesis

Random transposon mutagenesis in *F. spartansii* T16^T^ was performed using pHimarEm1 (Braun et al., 2005). Donor *E. coli* S17-1 λ *pir* cells with pHimarEm1 and recipient cells T16^T^ were grown to the log phase. Cells were collected by centrifugation at 3,800 rpm for 15 min. *F. spartansii* T16^T^ and *E. coli* cells were mixed at 1:1 ratios in CYE medium supplemented with Ca^2+^ and spotted onto CYE agar. After 24-h incubation at 28°C, the mixed cells were scraped from agar plates, washed once with CYE broth, and plated on the CYE agar containing erythromycin (100 μg/ml). Next, erythromycin transconjugants were transferred to PY2 agar (8%) for screening the spreading-deficient mutants. The gliding deficiency mutants were further observed for their motility on glass slides under microscopy. The pHimarEm1 insertion sites in the non-spreading mutants were identified using a published method (Braun et al., 2005). Briefly, genomic DNA was digested by XbaI overnight, purified with Qiagen PCR purification kit, and self-ligated. The ligation mixture was electroporated into *E. coli* Ec100D^+^ (Epicentre Technologies). Purified plasmid DNA from the Km-resistant colonies was sequenced using the primer Walker85 (TGGGAATCATTTGAAGGTTGG) and Walker86 (TCGGGTATCGCTCTTGAAGGG). The sequences were blasted against *F. spartansii* T16^T^ genome to characterize the insertion site. Growth between the selected mutants and WT was compared by determination of the OD600_nm_ after overnight culturing in PY2 broth (Braun et al., 2005).

### Hemolysin analysis

Hemolysin production in *F. spartansii* T16^T^ was examined by inoculating bacteria on Remel Blood Agar (Thermo Scientific, KS). Hemolytic activity was evaluated following incubation at 28°C for 48 h. The controls for α- and β-hemolysin producers were *Elizabethkingia meningoseptica* ATCC 13253 and *Staphylococcus aureus* MSU001, respectively (Chen et al., 2017).

### Antibiotic susceptibility testing

*Flavobacterium spartansii* isolates T16^T^ and S12 were tested for antibiotic susceptibility using the Kirby-Bauer disk diffusion method as previously described (Loch and Faisal, 2014b). In brief, cultures grown on Hsu-Shotts medium (48 h) were re-suspended in sterile 0.85% saline and adjusted to OD600_nm_ of 0.5 in a Biowave CO8000 Cell Density Meter (WPA Inc., Cambridge, UK). The bacterial suspension in duplicate was inoculated onto dilute Mueller-Hinton agar (Hawke and Thune, 1992) without 5% calf serum. Antibiotic-impregnated disks were stamped onto the medium and plates incubated at 22°C for 48 h, at which time the zones of inhibition were measured and means calculated. Antibiotics included polymyxin-B (PB; 300 iu), oxytetracycline (T; 30 μg), trimethoprim-sulfamethoxazole (SXT; 25 μg), erythromycin (E; 15 μg), ampicillin (AMP; 10 μg), florfenicol (FFC; 30 μg), penicillin G (P; 10 iu), and the vibriostatic agent O/129 (O129; 2,4-diamino,6,7-di-isopropyl pteridine; 10 μg). The cutoffs for sensitivity were as follows: *PB* < 8 mm resistant; 8–12 mm intermediate sensitivity; >12 mm sensitive; *T* < 15 mm resistant; 15–18 mm intermediate sensitivity; >18 mm sensitive; SXT < 11 mm resistant; 11–15 mm intermediate sensitivity; >15 mm sensitive; *E* < 14 mm resistant; 14–18 mm intermediate sensitivity; >18 mm sensitive; AMP < 12 mm resistant; 12–13 intermediate sensitivity; >13 mm sensitive; FFC < 16 mm resistant; 16–21 mm, intermediate sensitivity; >21 mm, sensitive; O/129, < 7 mm resistant, >7 mm sensitive (Whitman, 2004).

### Genome sequencing, assembly, and annotation

Next generation sequencing (NGS) libraries were prepared using the Illumina TruSeq Nano DNA Library Preparation Kit. Completed libraries were evaluated using a combination of Qubit dsDNA HS, Caliper LabChipGX HS DNA, and Kapa Illumina Library Quantification qPCR assays. Libraries were combined in a single pool for multiplexed sequencing and this pool was loaded on one standard MiSeq flow cell (v2) and sequencing was performed in a 2 × 250 bp paired end format using a v2, 500 cycle reagent cartridge. Base calling was done by Illumina Real Time Analysis v1.18.54 and the output of RTA was demultiplexed and converted to a FastQ format with Illumina Bcl2fastq v1.8.4 (Klein et al., 2013). NGS libraries were sequenced by Illumina Miseq paired-end sequencing technology at the Research Technology Support Facility (RTSF) at Michigan State University.

The reads were assembled using SPAdes (version 3.9.0. Gene annotation was carried out by NCBI Prokaryotic Genome Automatic Annotation Pipeline (PGAAP 3.3) (http://www.ncbi.nlm.nih.gov/genome/annotation_prok/). Initial prediction and annotation of open reading frames (ORF) and tRNA/rRNA gene prediction were carried out with Glimmer 3 through the Rapid Annotation using Subsystem Technology server (RAST).

### Bioinformatics

The functional categorization and classification for predicted ORFs were performed by RAST server-based SEED viewer (Aziz et al., 2008). The multi-drug resistance genes were predicted by Resistance Gene Identifier (RGI) tool implemented in CARD website (McArthur et al., 2013). Determination of phage elements was conducted in PHASTER (Arndt et al., 2016). Identification of genomic islands was performed in IslandViewer3 using *F. johnsoniae* genome as the reference (http://www.pathogenomics.sfu.ca/islandviewer). Prophage and Clustered Regularly Interspaced Short Palindromic Repeats (CRISPR) were predicted using the CRISPRfinder (Grissa et al., 2007). Detection and identification of virulence factors were carried out using MvirDB database (Zhou et al., 2007). Some virulence factors found in Bacteroidetes were manually compared among those fish pathogens and environmental isolates (Chen et al., 2005). For genome similarity assessment, digital DNA-DNA hybridization (dDDH) values were computed using web tool GGDC 2.1 (formula 2, identities/HSP length). Further, the average nucleotide identity (ANI) values were computed by ANI calculator (http://enve-omics.ce.gatech.edu/ani/).

The pan genome, core genome, and specific genes of *F. spartansii* were analyzed by comparison with 13 representative *Flavobacterium* using EDGAR 2.0 (Blom et al., 2016). The sizes of pan genome and core genomes were approximated using the core/pan development feature.

### Data deposition

This Whole Genome Shotgun project has been deposited at DDBJ/ENA/GenBank under the accession MIKE00000000. The version described in this paper is version MIKE00000000. The BioProject designation for this project is PRJNA341797. BioSample accession number is SAMN05728473.

## Results

### Genome features and phylogenic inference

The general genome features of *F. spartansii* compared to those representative *Flavobacterium*, and assembly statistics, are presented in Table 1. The assembly of *F. spartansii* produced 5.35 Mb of sequence across 29 contigs with an N_50_ of 515,812 bp, the longest sequence of 1,308,521 bp and a G+C content of 36% (Table 1). The genome was predicted to have at least 4,422 protein-coding genes and 130 RNA genes. Among the protein-encoding genes, 2,864 of them could be assigned a putative function, whereas 1,772 genes were predicted to encode hypothetical proteins. At least 2,119 proteins were assigned to 25 different functional categories with 368 subsystems using SEED subsystems by RAST analysis (Figure S1). The annotated genome had 112 genes responsible for bacteriocins (2 genes), resistance to antibiotics and toxic compounds (84 genes), and invasion and intracellular resistance (26 genes; Figure S1).

**Table 1:** The genome features of the selected *Flavobacterium* spp.

**Strains**	**Genome size (Mb)**	**CRISPR count**	**GC (%)**	**Predicted CDS**	**Total RNA**	**Enzyme count**
*F. psychrophilum* CSF259-93	2.90	1	32	2,520	198	650
*F. psychrophilum* JIP02/86	2.86	1	33	2,432	73	649
*F. psychrophilum* PG2	2.85	1	33	2,497	197	643
*F. columnare* ATCC 49512	3.16	3	31	2,646	89	674
*F. columnare* 94-081	3.30	1	31	2,847	89	705
*F. branchiophilum* FL-15	3.56	3	33	3,034	53	730
*F. spartansii* T16^T^	5.35	0	36	4,422	130	905
*Flavobacterium* sp. WG21	5.20	1	36	4,319	111	916
*F. hibernum* DSM 12611	5.28	2	33	4,510	132	1,009
*F. daejeonense* DSM 17708	4.20	0	35	3,622	89	873
*F. denitrificans* DSM 15936	4.80	2	34	4,121	133	957
*F. johnsoniae* UW101	6.10	1	34	5,017	82	1,003
*F. beibuense* F44-8	3.80	0	38	3,454	44	720

The ANI and dDDH values of representative *Flavobacterium* species are summarized in Table 2. *F. spartansii* and *Flavobacterium* sp. WG21 have an ANI value of 96.9% and a dDDH value of 72.7% (Table 2), suggesting that they are the same species according to the microbial taxonomy for species delineation (95 and 70% cut-off for ANI and dDDH, respectively) (Goris et al., 2007).

**Table 2:** Digital DNA-DNA Hybridization values (up, black font) and average nucleotide identity values (low, red font) amongst different *Flavobacterium* spp.

	***F. spartansii* T16^T^**	***F. psychrophilum* JIP02**	***F. psychrophilum* CSF259**	***F. psychrophilum* PG2**	***F. beibuense* F44-8**	***F. daejeonense* DSM 17708**	***F. columnare* ATCC 49512**	***F. columnare* 94-081**	***F. columnare* Pf1**	***F. johnsoniae* UW101**	***F. hibernum* DSM 12611**	***F. branchiophilum* FL-15**	***F. denitrificans* DSM 15936**	***Flavobacterium* sp. WG21**
***F. spartansii*** T16^T^														
***F. psychrophilum*** JIP02	**21.4**													
	**78.39**													
***F. psychrophilum*** CSF259	**21.2**	**98.9**												
	**78.39**	**99.91**												
***F. psychrophilum*** PG2	**21.4**	**99.6**	**99.1**											
	**78.44**	**99.93**	**99.93**											
***F. beibuense*** F44-8	**18.8**	**19.5**	**19.5**	**19.5**										
	**76.75**	**76.08**	**76.37**	**76.17**										
***F. daejeonense*** DSM 17708	**20.8**	**21.5**	**21.4**	**21.4**	**18.3**									
	**79.16**	**77.94**	**78.04**	**77.86**	**75.81**									
***F. columnare*** ATCC 49512	**23.2**	**21.5**	**21.3**	**21.4**	**20.8**	**21.3**								
	**78.52**	**76.85**	**78.52**	**76.72**	**76.95**	**77.96**								
***F. columnare*** 94-081	**21.6**	**21.1**	**21.1**	**21.1**	**20.3**	**20.6**	**42.6**							
	**77.74**	**77.02**	**76.9**	**77**	**78.18**	**77.97**	**90.71**							
***F. columnare*** Pf1	**22.3**	**21.7**	**21.7**	**21.6**	**21.1**	**21.6**	**95.9**	**42.7**						
	**78.03**	**77.22**	**78.03**	**77.08**	**76.92**	**77.59**	**99.62**	**90.83**						
***F. johnsoniae*** UW101	**23.9**	**22.0**	**22.1**	**22.1**	**19.6**	**21.3**	**22.6**	**22.7**	**22.9**					
	**81.96**	**78.61**	**78.9**	**78.71**	**76.02**	**78.92**	**77.53**	**78.47**	**77.84**					
***F. hibernum*** DSM 12611	**25.5**	**21.9**	**21.9**	**21.9**	**18.0**	**20.7**	**20.7**	**22.2**	**21.0**	**24.9**				
	**81.47**	**78.67**	**78.68**	**78.77**	**76.68**	**79.03**	**76.99**	**77.24**	**76.97**	**82.61**				
***F. branchiophilum*** FL-15	**21.6**	**22.1**	**22.2**	**22.4**	**20.2**	**21.1**	**20.9**	**22.1**	**21.6**	**21.9**	**21.2**			
	**77.94**	**78.31**	**78.43**	**78.45**	**76.46**	**78.3**	**77.21**	**78.19**	**77.32**	**78.7**	**78.06**			
***F. denitrificans*** DSM 15936	**23.4**	**20.7**	**20.8**	**20.7**	**18.8**	**20.8**	**20.5**	**22.1**	**20.9**	**25.9**	**24.6**	**21.3**		
	**81.47**	**77.84**	**77.92**	**77.9**	**76.72**	**79**	**77.05**	**77.21**	**76.96**	**83.43**	**82.14**	**77.55**		
***Flavobacterium*** **sp**. WG21	**72.7**	**21.5**	**21.2**	**21.5**	**18.6**	**20.7**	**23.1**	**22.4**	**23.2**	**23.9**	**25.7**	**21.8**	**23.5**	
	**96.93**	**78.57**	**78.49**	**78.48**	**76.83**	**79.28**	**77.99**	**77.58**	**77.95**	**81.66**	**82.84**	**77.95**	**81.45**	

### Gene repertoire of *flavobacterium*

**Figure 1:**
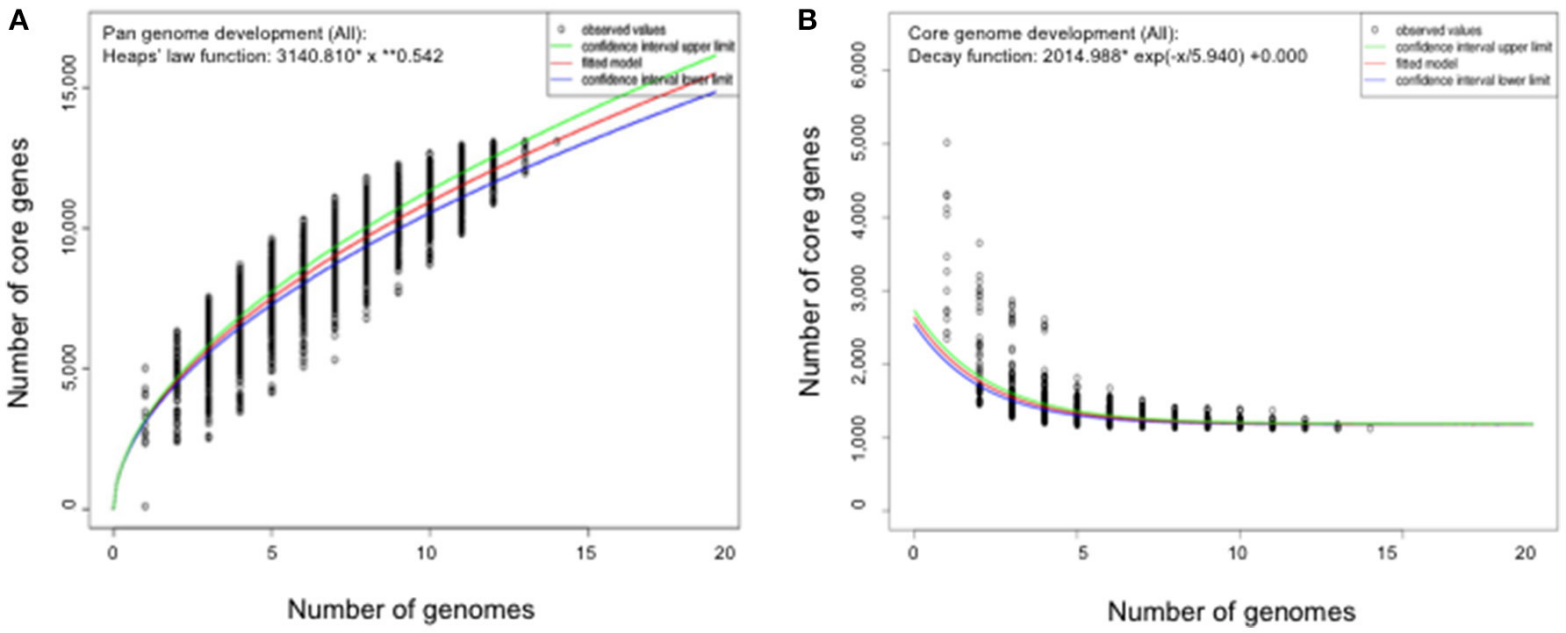
Pan and core genome evolution according to the number of sequenced *Flavobacterium* genomes. **(A)** Total number of genes (pan-genome) for a given number of genomes sequentially added. **(B)** Number of shared genes (core genome) as a function of the number of genomes sequentially added.

**Figure 2:**
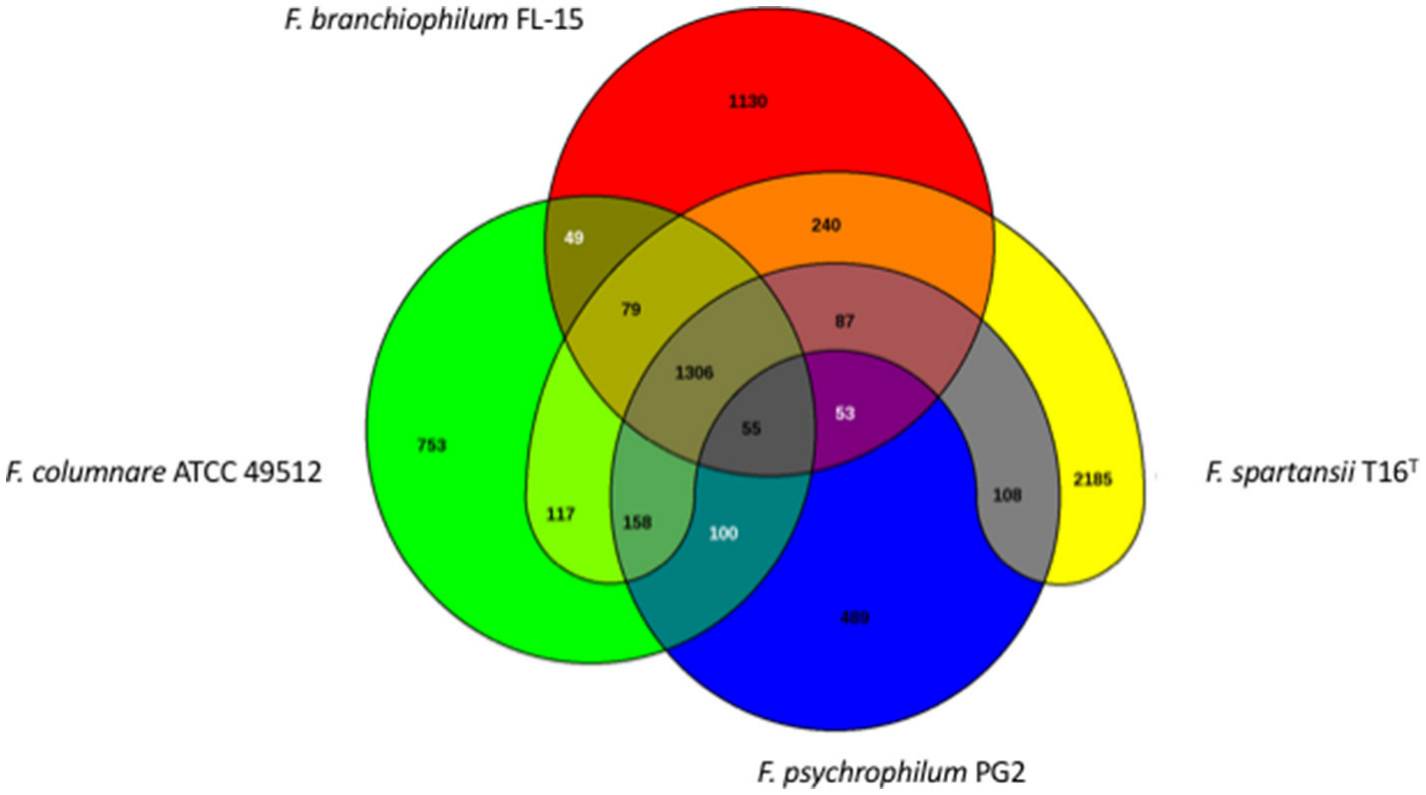
Venn diagram of shared and unique genes in the selected *Flavobacterium*. The unique and shared genome among the compared genomes were determined using the BLAST score ratio approach of EDGAR 2.0 with a cutoff of 30% (Blom et al., 2016).

The genome size for ubiquitous genes (core genome) or entire set of genome (pan-genome) amongst the selected *Flavobacterium* genomes was plotted against the number of genomes (Figure 1). The pan-genome plot showed that the power trend line had not reached the plateau (Figure 1), indicating that *Flavobacterium* possess an open pan-genome. Core genome analysis showed that the number of shared genes decreased with the addition of the input genomes and was predicted to converge against 1,182 (Figure 1). Actually, the core genome for the selected *Flavobacterium* in comparison was calculated to be 1,125 CDS per genome; thus, given the assumption that the prediction slightly overpredicts the core genome size, the current core genome represents the *Flavobacterium* genus quite well. *F. spartansii* shared at least 3,649, 3,201, 2,996, 2,774, 1,971, and 1,868 CDS with those environmental or opportunistic pathogenic *Flavobacterium* including *Flavobacterium* sp. WG211667 (87.2% of its total encoding genes), *F. hibernum* DSM 12611 (70.1%), *F. denitrificans* DSM 15936 (71.7%), *F. beibuense* F44-8 (69.6%) *F. daejeonense* DSM 17708 (68.2%), and *F. johnsoniae* UW101 (61.6%) (Figure 2). On the other hand, it shared up to 1,627, 1,651, and 1,712 CDS with the selected pathogenic *Flavobacterium* species such as *F. columnare* strains (~81%) and three *F. psychrophilum* strains (~75%) and *F. branchiophlium* (~71.3%).

### Antimicrobial resistance

Antibiotic susceptibility assays were conducted using the disk diffusion method (Table 3). Of the 8 tested antibiotics, *F. spartansii* T16^T^ was intermediately sensitive to trimethoprim-sulfamethoxazole, polymyxin-B, florfenicol, erythromycin as well as oxytetracycline, while it was resistant to ampicillin, penicillin G, 2,4-diamino and 6,7-di-isopropyl pteridine (Table 3). The resistome in strain S12 was similar to that in strain T16^T^. However, compared to strain T16^T^, strain S12 was more sensitive to low concentrations of erythromycin and immediate sensitive to ampicillin. Neither of the two *F. spartansii* isolates (Table 3) were completely sensitive to the two antibiotics (i.e., florfenicol and oxytetracycline) that are approved to treat certain flavobacterial diseases (e.g., bacterial coldwater disease caused by *F. psychrophilum*, and columnaris disease caused by *F. columnare*) in food fish (Bowker et al., 2016).

**Table 3:** Antibiotic susceptibility results for two *F. spartansii* isolates as determined via the Kirby-Bauer disk diffusion method.

**Isolate**	**SXT**	**PB**	**P**	**O129**	**FFC**	**AMP**	**E**	**T**
**T16**	I (11.5)	I (10.5)	R (6.0)	R (6.0)	I (16.0)	R (9.0)	I (18.0)	I (17.0)
**S12**	I (14.0)	I (11.0)	R (6.0)	R (6.0)	I (17.0)	I (12.0)	S (18.5)	I (16.5)

*R, resistant; I, intermediate sensitivity; S, sensitive. Number in parentheses denotes the mean diameter (in mm) of the zone of inhibition. SXT, trimethoprim-sulfamethoxazole (25 μg); PB, polymyxin-B (300 iu); P, penicillin G (10 iu); O129, 2,4-diamino,6,7-di-isopropyl pteridine (10 μg); FFC, florfenicol (30 μg); AMP, ampicillin (10 μg); E, erythromycin (15 μg); T, oxytetracycline (30 μg)*.

Both the RAST SEED subsystem and CARD were used to identify antibiotic resistance genes within the *F. spartansii* genome using the default settings. The predicted genes conferring resistance to aminocoumarin, mupirocin, chloramphenicol, fluoroquinolone, tetracycline, rifampin, and β-lactam are summarized in Table S1. Interestingly, up to 10 β-lactam resistance-related genes were found in the *F. spartansii* T16^T^ genome including those encoding the putative β-lactamases (8 copies), metallo-β-lactamases (1 copy) and penicillin-binding protein (1 copy). This observation is consistent with resistance to both penicillin and ampicillin in our antimicrobial tests (Table 3). Most β-lactamases in *F. spartansii* showed very low identity (<50%) to other fish pathogens (*F. psychrophilum* PG2*, F. columnare* and *F. branchiophilum* FL-15) (Table S1). However, a few lactamases have relatively high identity to those in *F. johnsoniae* and *Flavobacterium* sp. WG21. No erythromycin resistance related genes were detected in *F. spartansii*. More drug resistance genes and non-specific efflux pumps (5 hydrophobic/amphiphilic exporters, HAE1 family) possibly conferring antibiotic resistance are also conserved in *Flavobacterium* sp. WG21 and *F. hibernum* DSM 12611.

### Prophages

At least 5 prophages were predicted in *F. spartansii* by PHAST (Table S2). Among them, the prophage 1 (29.4 kb) consisted of 20 CDSs that encodes 12 proteins with known functions and 8 hypothetical proteins. Of interest, prophage 1 may be complete, as it consists of a phage tail, head, portal, integrase, lysin, terminase, and other component proteins involved in phage structure and assembly. The GC content (35.9%) in prophage 1 is very close to the average of GC in the whole genome (35.5%), indicating that prophage 1 may have been integrated into *F. spartansii* genome long ago. Unlike prophage 1, the remaining 5 prophages seem to be incomplete, as evidenced by the absence of a portion of phage structure proteins (Table S2). However, these regions carried genes encoding some important enzymes which may contribute to flavobacterial fitness under certain conditions. For example, prophage 2 had a DNA polymerase III beta subunit, methylase, NinG recombination, ATPase and Rec T protein, indicating this region may participate in DNA replication and modification. Prophage 3 carried many genes encoding enzymes related to polysaccharide (capsule precursor) metabolism; there were at least two 1,3-N-acetylglucosaminyltransferases, four glycosyl transferases, one GDP-D-mannose 4,6-dehydratase, one alpha-1,2-fucosyltransferase, one fucose synthetase, one dTDP-d-glucose 4,6-dehydratase, one L-fucosamine transferase and one UDP-N-acetylglucosamine 2-epimerase (Table S2). Prophage 4 may be linked to RNA metabolism because ribokinase-like domain-containing protein, RNA polymerase, ribonuclease H and putative ribonuclease 3 were organized as a cluster. Prophage 5 had genes encoding arsenate reductase and metallophosphoesterase, which possibly contribute to heavy metal resistance.

### Genomic islands and conjugative transposon (CTnFs)

**Figure 3:**
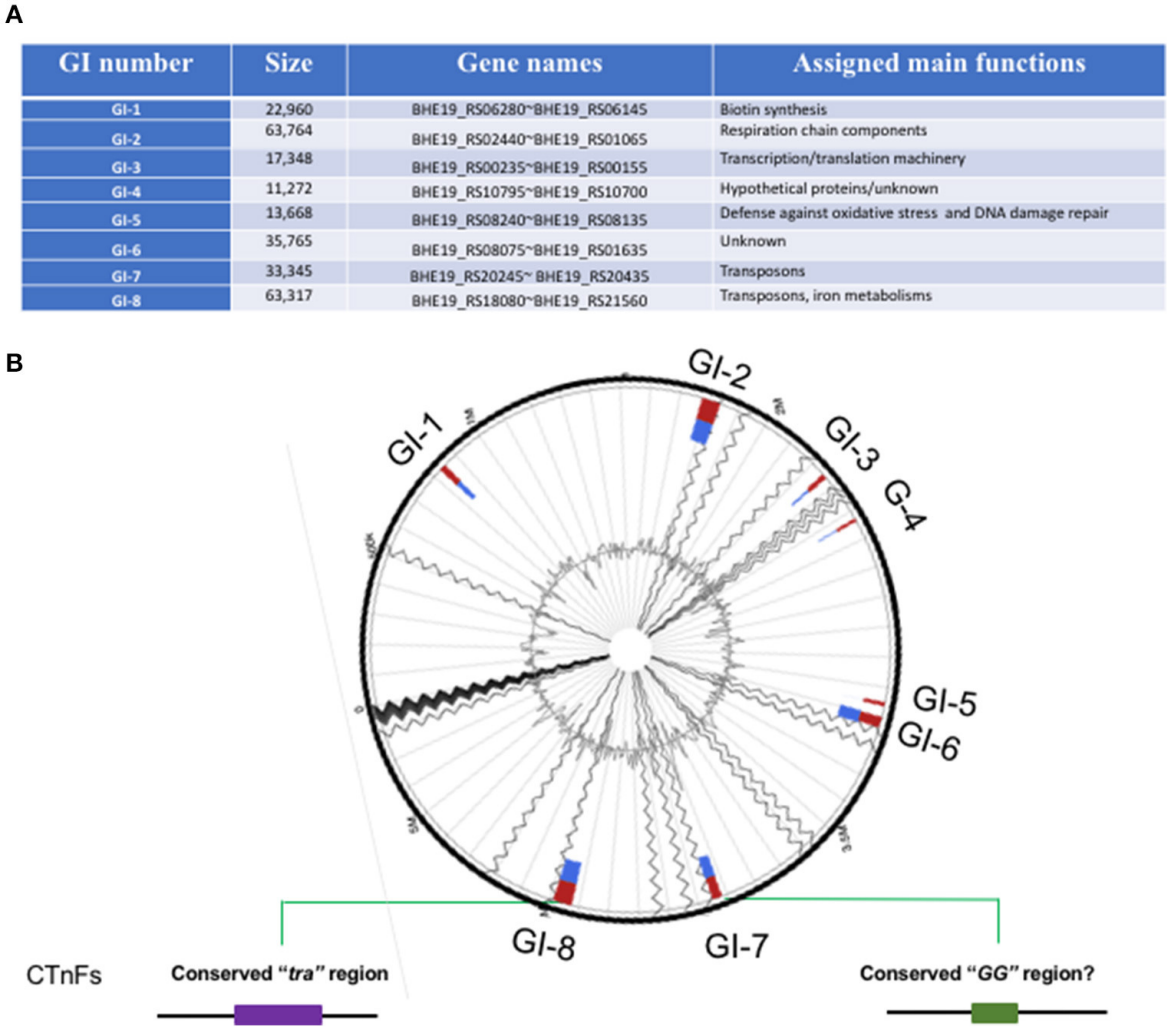
Genomic islands in *F. spartansii* T16^T^. **(A)** The 8 genomic islands were predicted by Islandviewer. **(B)** The schematic of GI-associated features. The relative locations of the 8 GIs were shown in the predicted genome. The conjugation protein genes belonging to “*tra*” and “*GG*” in CTnFs were centered in the GI-7 and GI-8 regions, respectively.

At least 8 genomic islands (GIs) were identified in *F. spartansii* (Figures 3A,B and Table S3) by both IslandPick and IslandPath-DIMOB methods (Nagamatsu et al., 2015). The GI size ranged from 11.3 to 63.8 Kb (Figure 3A). Genes encoding virulence factors, toxins, DNA metabolism, transposases, regulators, modification and restriction systems and resistance to antibiotics occurred in these GIs (Figure 3 and Table S3), indicating that *F. spartansii* T16^T^ possibly acquired these genes, thereby forming GIs favoring adaptation to diverse environments (see below). Among the 8 GIs, GI-8 had the largest size (63.7 Kb). Notably, GI-8 has many genes encoding components of a conjugative transposon in Bacteroidetes (here, named CTnFs), e.g., *traN, traM, traK, traJ, traI*, and *traG*. Moreover, the remaining components of this large conjugative transposon CTnFs can be found in GI-7 (33.3 kb) including *traD, traC, traE, traF, traG, traI, traJ, traK, traM, traI*, and *traQ* genes (Table S3). We further examined the surrounding regions around GI-7 and found the *traA* and *traC* genes which are located upstream of *traD* (Table 4). The distribution spectrum of the CTnFs-like conjugative transposons were investigated by searching the conserved transfer protein gene *traG* (a signature gene for conjugative transposons) among the selected *Flavobacterium* species. No *traG* gene sequences (cutoff 50%) were found in *F. psychrophilum, F. columnare*, and *F. branchiophilum* FL-15. The same results were obtained when using other *tra* genes to search against their genomes, indicating that the conjugative transposon is absent in these fish pathogens. By contrast, the similar conjugative transposons were observed in *F. johnsoniae, Flavobacterium* sp. W21, *F. hibernum* DSM 12611, *F. daejeonense* DSM 17708, *F. denitrificans* DSM 15936, and *F. beibuense* F44-8 (Table 4).

**Table 4:** Transfer proteins in the selected *Flavobacterium* spp.^*^.

**Genome island**	**Locus (BHE19_RS)**	**Gene product**	***F. spartansii* T16^T^**	***F. psychrophilum* CSF259-93**	***F. psychrophilum* JIP02/86**	***F. psychrophilum* PG2**	***F. columnare* ATCC 49512**	***F. columnare* 94-081**	***F. branchiophilum* FL-15**	***Flavobacterium* sp. WG21**	***F. hibernum* DSM 12611**	***F. daejeonense* DSM 17708**	***F. denitrificans* DSM 15936**	***F. johnsoniae* UW101**	***F. beibuense* F44-8**
GI-7	20235	TraA	100	-	-	-	-	-	-	-	51	78	52	77	-
	20240	TraB	100	-	-	-	-	-	-	-	36	66	38	62	-
	20245	TraD	100	-	-	-	-	-	-	-	71	71	71	61	-
	20255	TraF	100	-	-	-	-	-	-	-	40	79	77	75	-
	20215	TraG	100	32	32	32	-	-	-	53	88	88	87	83	50
	20260	TraG	100	-	-	-	-	-	-	-	55	90	91	84	54
	20270	TraJ	100	-	-	-	-	-	-	-	40	85	85	85	42
	20275	TraK	100	-	-	-	-	-	-	-	46	88	90	86	46
	20290	TraM	100	-	-	-	-	-	-	-	35	62	66	53	35
	20295	TraN	100	-	-	-	-	-	-	-	32	72	70	51	35
	20300	TraO	100	-	-	-	-	-	-	-	-	67	71	-	-
	20305	TraQ	100	-	-	-	-	-	-	-	-	64	69	-	-
GI-8	17685	TraG	100	32	32	32	-	-	-	77	75	51	51	96	74
	17810	TraI	100	-	-	-	-	-	-	-	77	21	20	98	64
	17825	TraJ	100	-	-	-	-	-	-	-	77	42	43	97	72
	17830	TraK	100	-	-	-	-	-	-	-	83	46	45	98	75
	17840	TraM	100	-	-	-	-	-	-	-	54	31	35	97	49
	17845	TraN	100	-	-	-	-	-	-	-	50	32	32	93	43

* *The numbers representing amino acid identity (%). “-” indicates that there is no hit output*.

GI-2 had many genes encoding respiratory chain components such as cytochrome C subunits, cytochrome P450, quinol:cytochrome C oxidoreductase, and several other membrane proteins (Table S3). GI-2 had the predicted virulence factor *VirJ*, possibly participating in the Type IV secretory pathway (Table S3). Immediately downstream of GI-2, there are genes involved in the gliding motility (*Rem*B and *Spr*A). Another large genomic island, GI-6, contained a gene cluster encoding DNA repair proteins and enzymes such as MsrA (preventing oxidative damage), NTP pyrophosphohydrolases, chaperone protein DnaK, deoxyribodipyrimidine photolyase (DNA repair enzyme), DNA alkylation repair enzyme, peroxiredoxin, and DNA-damage-inducible protein D (Table S3). On the same GI, there were transcriptional regulators from GntR, HxlR, and AraC families, showing that the GI-1 may be involved in the defense against oxidative stress and DNA damage repair. Compared to the above 4 GIs, the remaining ones (GI-1, GI-3, GI-4, and GI-5) were smaller (Table S3).

### Prediction of virulence factors

When the proteome of *F. spartansii* T16^T^ was used in a BLAST search against MvirDB database, 1,001 virulence factors were predicted (cutoff E-10) (Table S4). Further, 133 putative virulence proteins were found to be highly conserved in *F. spartansii* T16^T^ (cutoff 50%) (Table S4). Virulence factor matches in the MvirDB database include well-known proteases, catalase/peroxidase HPI, conjugal transfer proteins (4 Tra components), molecular chaperones (GroEL, DnaJ, and DnaK), translation initiation factor IF-1, transcription regulation factors, antimicrobial resistance proteins, LPS biosynthesis protein WbpP, biopolymer transporter (ExbD and SusD/RagB), ribosomal proteins, carbon and nitrogen metabolism related proteins as well as iron-binding proteins (Table S4).

**Figure 4:**
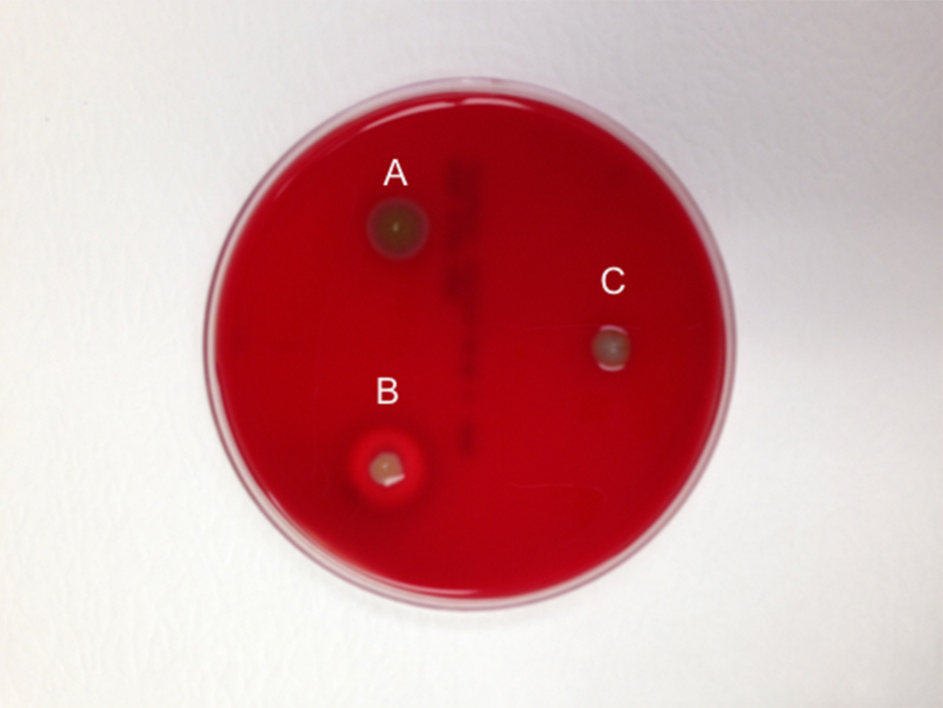
Demonstration of the hemolytic activity in *F. spartansii*. The hemolytic activity on sheep blood agar plate was observed after 48-h incubation. **(A)** *F. spartansii*, **(B)** *S. aureus* MSU001, the β-hymolysin control, and **(C)** *E. meningoseptica* ATCC 13253, α-hemolysin control.

At least three hemolysin genes were present in *F. spartansii* (Table 5), which complements observations that strain T16^T^ has *in vitro* hemolytic activity (Figure 4). When grown on sheep's blood agar plate for 48 h, colonies of *F. spartansii* T16^T^ appeared greenish, suggesting α-hemolytic activity (Figure 4). Moreover, many genes encoding proteolytic enzymes were discovered in the genome of *F. spartansii* T16^T^ (Table 5). Further, the amino sequence of a collagenase (BHE19_RS20565, peptidases U32) was conserved in most of the selected *Flavobacterium* (Table 5).

**Table 5:** Proteases and hemolysins in *Flavobacterium* spp.^*^.

**Locus (BHE19_RS)**	**Gene product**	***F. spartansii* T16^T^**	***F. hibernum* DSM 12611**	***F. johnsoniae* UW101**	***Flavobacterium* sp. WG21**	***F. psychrophilum* JIP02/86**	***F. branchiophilum* FL-15**	***F. columnare* ATCC 49512**	***F. psychrophilum* CSF259-93**	***F. psychrophilum* PG2**	***F. denitrificans* DSM 15936**	***F. beibuense* F44-8**	***F. daejeonense* DSM 17708**	***F. columnare* 94-081**
**PROTEASES**														
04620	Metalloprotease RseP	100	86	86	99	43	41	41	43	43	69	67	79	42
16755	Peptidase M1	100	88	90	99	47	45	44	47	47	91	46	67	44
22130	Peptidase M43	100	85	85	100	58	65	67	58	58	84	35	-	68
09105	Peptidase S8	100	78	72	99	55	49	51	55	55	70	54	55	51
04735	Aminopeptidase	100	96	94	100	74	54	70	74	74	94	68	-	71
20565	Collagenase U32	100	98	95	99	69	85	83	69	69	97	-	69	82
**HEMOLYSINS**														
15730	Hemolysin	100	96	93	100	75	78	75	75	75	93	78	82	75
03430	Hemolysin	100	92	88	99	25	72	28	26	26	88	64	80	27
04015	Hemolysin D	100	93	86	99	24	-	76	24	24	87	-	70	-

* *The numbers representing amino acid identity (%). “-” indicates that there is no hit output*.

### Gliding machinery and random mutation analysis

In *F. spartansii*, at least 21 genes encoding proteins associated with gliding motility were identified, including *gldA, gldB, gldC, gldD, gldE, gldF, gldG, gldH, gldI, gldJ, gldK, gldL, gldM, gldN, SprA, SprB, SprC, SprD, SprE, SprF, and SprT* (Table 6). The motility protein sequences were conserved among these *Flavobacterium* genomes except GldE, SprB, and SprC (Table 6). Genes encoding PorV and RemA also existed in *F. spartansii* T16^T^ (Table 6). Therefore, *F. spartansii* possesses the minimal requirement of the full function of T9SSs (also important for gliding) consisting of GldK, GldL, GldM, GldN, SprA, SprE, and SprT (McBride and Nakane, 2015).

**Table 6:** Comparation of gliding machinery in selected *Flavobacterium* spp.^*^.

**Gene product**	**Locus (BHE19_RS)**	***F. spartansii* T16^T^**	***F. psychrophilum* JIP02/86**	***F. psychrophilum* CSF259-93**	***F. psychrophilum* PG2**	**F. beibuense F44-8**	***F. daejeonense* DSM 17708**	***F. columnare* ATCC 49512**	***F. columnare* 94-081**	***F. johnsoniae* UW101**	***F. hibernum* DSM 12611**	***F. branchiophilum* FL-15**	***F. denitrificans* DSM 15936**	***Flavobacterium* sp. WG21**
**GENE PRODUCTS INVOLVED IN GLIDING**														
gldA	02885	100	77	77	77	77	83	73	73	92	94	77	93	99
gldB	20835	100	55	55	55	49	65	49	50	88	89	57	88	99
gldC	20840	100	83	83	83	76	85	79	79	93	96	82	94	100
gldD	02790	100	59	59	59	57	74	61	61	88	95	70	90	100
gldE	02795	100	23	23	23	67	80	26	26	90	90	29	88	100
gldF	01675	100	75	75	75	76	81	68	65	92	93	74	91	99
gldG	01670	100	61	61	61	63	77	59	58	88	92	61	87	99
gldH	05785	100	57	57	57	60	77	56	57	93	91	59	92	100
glaI	09295	100	56	56	56	57	65	53	52	80	84	57	76	99
gldJ	02660	100	70	70	70	68	79	69	68	94	96	73	92	100
gldK	21030	100	76	76	76	77	86	76	76	96	97	80	97	100
gldL	21035	100	87	87	87	76	83	74	73	94	89	72	96	99
gldM	21040	100	68	68	68	51	79	65	65	94	86	63	88	100
gldN	21050	100	64	64	64	50	70	63	62	88	88	61	87	99
**GENE PRODUCTS INVOLVED IN SPREADING**														
sprA	00945	100	64	64	64	60	72	57	58	88	91	60	88	99
sprB	05460	100	39	37	39	40	38	33	39	37	37	39	37	98
sprC	05450	100	36	36	36	34	43	32	34	61	59	-	60	99
sprD	05455	100	43	43	43	35	55	42	39	80	83	-	82	99
sprE	05120	100	49	49	49	47	59	48	47	81	83	45	78	99
sprF	05455	100	43	43	43	35	55	42	39	80	83	-	82	99
sprT	03165	100	57	57	57	67	77	56	59	88	92	61	87	100
**OTHERS INVOLVED IN T9SS SECRETION**														
porV	02670	100	68	68	68	68	71	64	66	87	92	65	83	99
remA	22245	100	40	40	40	42	50	31	41	48	74	36	48	83

* *The numbers representing amino acid identity (%). “-” indicates that there is no hit output*.

**Figure 5:**
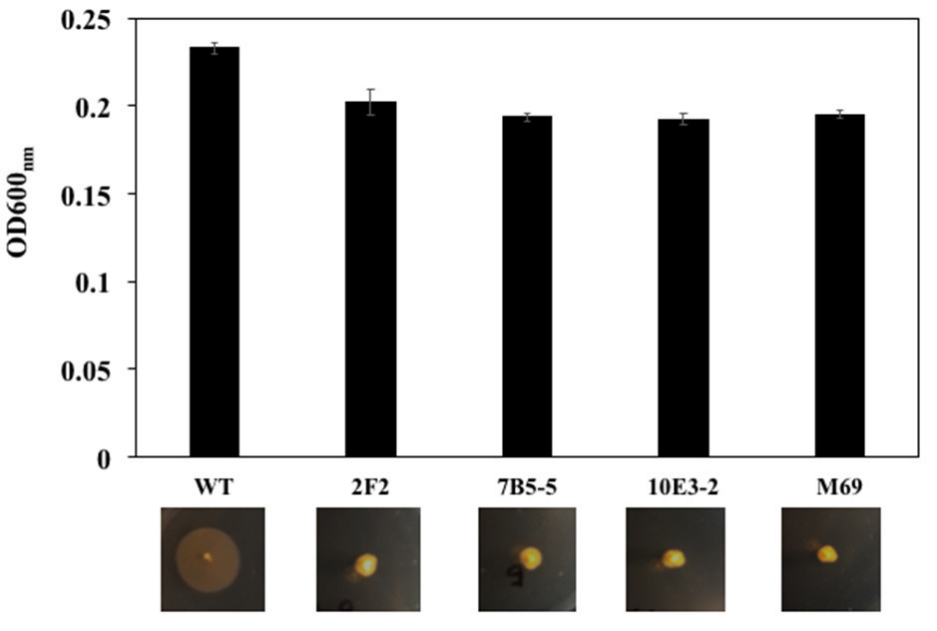
Comparation of the cell growth in PY2 broth and gliding motility on the PY2 agar between the WT and mutants. Transposon pHimarEm1 was introduced into *F. spartansii* T16^T^ and erythromycin-resistant conjugants were sub-cultured in PY2 broth. The cell growth was compared by determining the OD600nm after overnight culture (up panel). The motility was evaluated by culture the cells on PY2 agar after 48 h (low panel).

A transposon used for random mutagenesis in flavobacteria, pHimarEm1, was functional in *F. spartansii* T16^T^. Out of 2,000 conjugants, at least 4 mutants were recovered with impaired ability to spread on PY2 agar or glide on the surface of glass slides (Figure 5). For mutant 2F2-2, the transposon inserted 6-bp upstream of the ATG start codon of a gene encoding a hypothetical protein (BHE19_RS13930). For mutants 7B5-5, the insertion of pHimarEm1 transposon was 14 bp downstream of the ATG start codon of an aconitase gene (BHE19_RS00150). In mutant 10E3-2 the transposon inserted 1,380 bp downstream of the ATG start codon of the *gldM* gene (BHE19_RS21040). For the mutant M69, the transposon inserted at 67 bp downstream of the ATG start codon of *EpsM* gene (BHE19_RS15305). The difference in biomass determination was negligible between the WT and mutants, indicating that these gliding genes were not critical for cell growth in PY2 broth (Figure 5).

## Discussion

Our results demonstrated that strain T16^T^ possesses some virulence factors that are similar to those previously reported in other Bacteroidetes, including metalloproteases, and hemolysins (Table 5). Moreover, the type 9 secretion system (T9SS) also occurs in T16^T^, which may contribute to secreting proteins that may be involved in virulence (Table 6). Nevertheless, our results also show that T16^T^ has some virulence factors and antimicrobial genes absent in well-known fish pathogens (i.e., *F. columnare, F. Branchiophilum*, and *F. psychrophilum*; Table 5). The GC content in strain T16^T^ (36%) was higher than in *F. columnare* ATCC 49512 (31%), *F. branchiophilum* FL-15 (33%), and *F. psychrophilum* JIP02/86 (33%), indicating their diverse genome evolution under differential selection (Lassalle et al., 2015). Among the selected flavobacteria, the genome size of *F. spartansii* T16^T^ (5.35 Mb, next to *F. johnsoniae*) was remarkably larger than that in typical fish pathogens such as *F. columnare* ATCC 49512 (3.16 Mb), *F. branchiophilum* (3.56 Mb), and *F. psychrophilum* JIP02/86 (2.86 Mb). Genome size in opportunistic pathogens is typically larger than in virulent strains because more functional genes are required for their complicated living styles and diverse environments. Kolton et al. (2013) reported the genome size in *Flavobacterium* belonging to the terrestrial clade is around 40% larger than within the aquatic clade (Kolton et al., 2013).

Genomic islands often drive microbial evolution, increase fitness, and contribute to species diversity (Jackson et al., 2011). It is interesting that a large conjugative transposon (CTnFs) spanning at least two large GIs (GI-7 and GI-8) was found in *F. spartansii* T16^T^. Such conjugative transposons are widespread in *Bacteroides, Prevotella*, and *Porphyromonas* (Franco, 2004; Naito et al., 2011; Gorenc et al., 2012). Here, we extended the distribution profiles of conjugative transposons to some flavobacterial members including *F. spartansii, F. johnsoniae, F. hibernum, F. denitrificans, F. beibuense*, and *F. daejeonense* (Table 4). Gene content and organization of CTnFs-like conjugative transposons in *F. spartansii* mimic those reported in transposons CTn3-Bf and CTnB_1_4 of *B. fragilis* YCH46 and *P. bryantii* B_1_4T (Gorenc et al., 2012). One of the most intriguing features of conjugative transposon is presence of two conserved regions called “*tra*” and “GG” (Gorenc et al., 2012). However, CTnFs-like transposons have not been reported in *F. columnare, F. branchiophilum* and *F. psychrophilum* species based on marker gene search (*traG*), nor were CTnFs genes detected in *Flavobacterium* sp. W22 (Table 4) though its phylogeny placement is very close to *F. spartansii* T16^T^. The reason for the absence of CTnFs-like transposons in typical *Flavobacterium* pathogens remains unknown. The size of conjugative transposon is generally large (100–210 kb in *Bacteroides*) but varies considerably amongst species. CTnB_1_4 in *P. bryantii* B_1_4T is around 212 kb with a spacer (49.1 kb) where a large sugar utilization gene cluster locates (Gorenc et al., 2012). This conjugative transposon may promote the ability to scavenge the glycans *in vivo* in this oral cavity-adapted symbiont (Matsui et al., 2000). The accurate size, full contents and physiological roles in the CTnFs remain unclear in free-living isolates or opportunistic pathogens (see next). However, many functional gene products were predicted between/around the two regions (Gorenc et al., 2012). While the conjugative transposon is activated and mobilized, it frequently carries over its close regions to the new hosts (Juhas et al., 2009). Thus, the conjugative transposons may have important implications for spreading virulence factors, metabolism genes and some resistance genes (Burrus and Waldor, 2004; Juhas et al., 2009). Furthermore, the transfer efficiency of CTnDOT/ERL conjugative transposons in *Bacteroides* (also found in other Bacteroidetes members such as *Prevotella*), improved at least 1,000-fold when a low concentration of tetracycline was present in the culture (Jeters et al., 2009). The phenomenon indicates that antibiotics in the environment (such tetracycline) not only provide selection pressure for Bacteroidetes to acquire foreign antimicrobial genes, but also promote spreading other antibiotic genes (e.g., erythromycin gene transfer in *Bacteroides*) or other genetic elements (Shoemaker et al., 2001). Because the conjugative transposons present in *Flavobacterium* strains are very similar to CTnDOT, one can infer that horizontal transfer of flavobacterial genes involved in antibiotic resistance and/or virulence factors is very likely in their hosts and native habitats.

There are multiple antibiotics and chemotherapeutic compounds that are currently approved for use in foodfish or can be utilized under an investigational new animal drug (INAD) status (https://www.fws.gov/fisheries/aadap/aquaculture.html). Thus, generating more antibiotic sensitivity data for *F. spartansii* is crucial for guiding further research toward additional antibiotic approvals. *F. spartansii* T16^T^ is intermediate in susceptibility to the permitted antibiotics in the USA aquaculture industry, namely oxytetracycline, sulfadimethoxine and florfenicol. One of the striking features in resistome analysis of T16^T^ is that there are at least 10 β-lactam resistance-related genes. Putative β-lactamases (8 copies), metallo-β-lactamases (1 copy) and penicillin-binding proteins (1 copy) possibly confer resistance to penicillin and ampicillin (Table S1). Resistance toward third-generation cephalosporins (data not shown) by class C beta-lactamases seen in *F. spartansii* would hamper treatment of infection by *Flavobacterium* species. The concern is that, as a reservoir of extended-spectrum class C beta-lactamases, *F. spartansii* may spread these resistance gene(s) to other flavobacterial pathogens in the future. Extensive antibiotics application in the aquaculture industry may account for the multidrug resistance profile seen in resistance genes of *F. spartansii* (Kristiansson et al., 2011). It is interesting that two genes conferring tetracycline resistance were found in *F. spartansii* (as in several other *Flavobacterium* species) while disc analysis showed that bacterial cells were intermediate in susceptibility to tetracycline (Table 3). A tetracycline resistance (*tetX*) gene in transposons Tn4351 and Tn4400 did not confer resistance on anaerobically-grown *Bacteroides fragilis* while it functioned in aerobically-grown *E. coli* (Speer and Salyers, 1990; Speer et al., 1991). Gene *tetX*, located inside the conjugative transposon, may be acquired from other bacteria (exemplified by the GC ratio compared to the average ratio in the genome) during the transposon mobilization process. Expression of *tetX* may be problematic in *F. spartansii* because it is known that the transcriptional and translational initiation signals from Proteobacteria or the Gram-positive bacteria are not recognized well in Bacteroidetes (Chen et al., 2007a). Drug resistance mechanisms in bacteria are multifactorial, and can involve enzymatic degradation of the drugs, direct extrusion of the drug from the cells through efflux pumps, or alteration/mutation of ribosomal binding sites (Nikaido, 2009). The multidrug resistance efflux pumps predicted in *F. spartansii* and other flavobacteria are possibly involved in antimicrobial resistance, which does not manifest in other antibiotic degradation genes (Nikaido, 2009; Sun et al., 2014). This situation is cause for concern, and warrants more stringent surveillance in the use of antibiotics, as well as the resultant antibiotic resistance in clinically important bacterial species. Whole genome sequence of *F. spartansii* will be useful in future studies to determine antimicrobial resistance and virulence attributes as well as mechanisms that enhance its environmental or host fitness.

Besides the conjugative transposons and genomic islands, bacteriophages and phage-like genetic elements occupy a great amount of bacterial genome space. Bacteriophage-mediated transduction is an important contributor to spreading antimicrobial resistance and metabolism genes by the horizontal gene transfer mechanisms (Dutta and Sarkar, 2015). At least 5 prophages were predicted in *F. spartansii* T16^T^ (Table S2). Only one of them (prophage 1) seems to be complete. However, the other prophages should not just be regarded as inactive because they may share machinery (e.g., structure proteins or replication enzymes) originating from prophage 1 or from the host, thus they can successfully assemble, pack and activate (Drulis-Kawa et al., 2012). Genes encoding important enzymes involved in capsule precursor biosynthesis and heavy metal resistance were found within the regions of the predicted prophage genomes, indicating that it is possible that these prophages possibly shape the bacterial genome evolution (Table S2). However, how these prophages influence the host behavior and virulence remains unexplored in flavobacteria. Indeed, prophages or phage-like genetic elements are not as prevalent in flavobacterial pathogen genomes as those in *F. spartansii* T16^T^; e.g., most of *F. psychrophilum* strains (such as 950106-1/1, JIP02/86, MH1, PG2, and 5) carry only one prophage (named 6H); moreover, there are only 1 and 3 incomplete phage clusters (no complete one) identified in *F. columnare* 94-081 and *F. columnare* ATCC 49512, respectively. Similarly, Touchon et al. reported that there were no incomplete prophage elements in *F. branchiophilum* FL-15 (Touchon et al., 2011). This may be partially explained due to the presence of multiple CRISPR loci in *F. psychrophilum* (1~2 loci) (Castillo et al., 2016), *F. columnare* (at least 3 loci) (Kayansamruaj et al., 2017), and *F. branchiophilum* FL-15 (at least 3 loci) (Touchon et al., 2011; Barrangou and Marraffini, 2014; van Houte et al., 2016). It is interesting that we did not detect any CRISPR elements in *F. spartansii* T16^T^, which may coincide with presence of prophages, genomic islands, and conjugative transposons in its genome. CRISPR/Cas, the prokaryotic immune system, defends the foreign DNA invasion (plasmids, phages and transposons) and may participate in regulating stress gene response and controlling bacterial virulence (Louwen et al., 2014; Laanto et al., 2017).

Prediction of virulence factors in this study contributes to our understanding of flavobacterial pathogenesis mechanisms as well as *Flavobacterium*/host interactions. The predicted virulence factors have good homology with those discovered in other well-known pathogenic flavobacteria (Touchon et al., 2011; Kumru et al., 2017). For example, *F. spartansii* T16^T^ has the capability to digest animal erythrocytes with α-hemolytic activities, consistent with three hemolysin genes predicted in *F. spartansii* as virulence factors (Table 5). Hemolysins are well-documented cytolytic toxins that are important for animal pathogenesis process such as sepsis and tissue damage (Portnoy et al., 1988; Los et al., 2013). In *F. columnare* strain 94-081, disruption of one of the hemolysin genes (AWN65_RS11020) decreased fish mortality 15%, indicating that it contributes to virulence (Kumru et al., 2017). Moreover, this observation also showed that additional hemolysin(s) may be necessary to have full virulence against fish (Kumru et al., 2017). In addition, *F. spartansii* T16^T^ has the thiol-activated cytolysin (TACYs) that possibly forms pores on the erythrocyte membrane (Morgan et al., 1996). Moreover, TACYs can also lead to the triggering signaling pathways in host cells, as is the case for listeriolysin O (LLO) (Hamon et al., 2012). LLOs induce a cytokine response by functioning as a pleiotropic pseudocytokine/chemokine, thus strongly influencing the course of infection (Baba et al., 2002). Protein sequences of a collagenase (peptidases U32) in strain T16^T^ are conserved amongst the selected *Flavobacterium* strains (Table 5). Nakayama et al. (2016) found that the collagenase gene (*fpcol*) contributed to mortality in the Ayu (*Plecoglossus altivelis*) (Nakayama et al., 2016). Further, they also reported that the expression of *fpcol* was partially repressed by calcium or gelatin, showing that collagenase was necessary when the bacterial cells adhered to fish surface and initiated the invasion process (Nakayama et al., 2016). *F. spartansii* T16^T^ has several metalloproteases (Table 5), which may facilitate bacterial dispersion and tissue damage. Conjugal transfer proteins (4 Tra components) as virulence factors probably allow these genomic elements to transfer from or to other bacteria (Vogel et al., 1998).

Fourteen *gld* and seven *spr* genes participating in the gliding motility, respectively, were found in *F. spartansii* T16^T^ genome (Table 6). The amino acid sequences of the gliding and spreading proteins are highly conserved among the selected flavobacteria except GldE (Table 6). *GldE* (cutoff 50%) seem to be absent in the genomes of *F. psychrophilum* and *F. columnare*, indicating that they are not critical for gliding in these two *Flavobacterium* species. In *F. johnsoniae*, the gliding function of *gldE* can be replaced by *gldB* (Hunnicutt and McBride, 2001). Seven spreading protein encoding genes (SprA, SprB, SprC, SprD, SprE, SprF, and SprT) were found in *F. spartansii* T16^T^ genome (Table 6). Most of the gene products (except SprB) show good homology to those in *F. johnsoniae* and *F. hibernum* DSM 12611, *F. denitrificans* DSM 15936 and *Flavobacterium* sp. WG21 though some of them (SprB and SprC) have the relative low identity to those in fish pathogens *F. psychrophilum* and *F. columnare* (Table 6).

Genetic amenability of *F. spartansii* T16^T^ was exemplified by obtaining several gliding-deficient mutants using a transposon mutagenesis method. It is not surprising that we found one of the motility mutants had disruption of the gliding gene *gldM*. Disability of *gldM* gene expression in *F. johnsoniae* and *Cellulophaga algicola* caused gliding deficiency on the slide or agar surfaces, as well as failure of the delivery of SprB to the cell surface, and secretion of many cell surface and extracellular proteins (Braun et al., 2005; Zhu and McBride, 2016). Moreover, disruption of some gliding genes in flavobacteria led to attenuated virulence or biofilm formation (Álvarez et al., 2006; Sato et al., 2010). Decreasing virulence in gliding mutants may be due to the disruption of the T9SSs function (Sato et al., 2010). Diverse CTDs involved in protein secretion were recently characterized in *F. johnsoniae* (Chen et al., 2007b; Kulkarni et al., 2017). Proteins secreted through T9SSs possess two critical signals: the first one is the N-terminal signal peptides that are required by the Sec system to export outside the cytoplasmic membrane; and the second one is the carboxy-terminal domains (CTDs) that are needed by the T9SSs to secrete through outer membranes (McBride and Zhu, 2013; de Diego et al., 2016; Kulkarni et al., 2017). However, more research is needed to provide more evidences for virulence factors secreted through T9SSs in these flavobacterial pathogens. Furthermore, three putative gliding motility genes encoding the hypothetical protein belonging to the HCP-like family, the aniconase involved in TCA cycle for energy production, and a polysaccharide synthesis component. The complementation of these mutants is warranted to further confirm the gene function(s) involved in gliding motility.

## Author contributions

SC and EW conceived the study and participated in its design and coordination. SC performed the experiments, whole genome sequencing, annotation, and comparative analysis. JB contributed to the genome analysis. SC, TL, MF, and EW wrote the manuscript. All authors have read and approved the manuscript.

### Conflict of interest statement

The authors declare that the research was conducted in the absence of any commercial or financial relationships that could be construed as a potential conflict of interest.
