# Supplementary material for: The Emerging Fish Pathogen Flavobacterium spartansii Isolated from Chinook Salmon: Comparative Genome Analysis and Molecular Manipulation
Source: Front Microbiol. 2017 Nov 30;8:2339. doi: 10.3389/fmicb.2017.02339 (PMC5714932; doi:10.3389/fmicb.2017.02339)
Supplement: Supplementary file 1 [file Table1.docx]

| **Table S1. Comparation of the antimicrobial resistance genes among the selected flavobacteria** | | | | | | | | | | | | | | | | |
| --- | --- | --- | --- | --- | --- | --- | --- | --- | --- | --- | --- | --- | --- | --- | --- | --- |
| **Antibiotics** | **Locus ID** | **Function** | **Identity (%)** | | | | | | | | | | | | |  |
|  |  |  | ***Fs*** | ***Fp*JIP02/86** | ***Fp*CSF259-93** | ***Fp*PG2** | ***Fb*F44-8** | ***Fd*DSM17708** | ***Fc*ATCC49512** | ***Fc*94-081** | ***Fj*UW101** | ***Fh*DSM12611** | ***Fb* FL-15** | ***Fd*DSM 15936** | ***Fs* WG21** |  |
| Isoniazid |  | catalase-peroxidase | 100 | 68 | 68 | 68 | 66 | 70 | 59 | 59 | 65 | 0 | 65 | 86 | 99 |  |
| Aminocoumarin | BHE19_RS00500 | alanyl-tRNA synthetase | 100 | 71 | 71 | 71 | 75 | 74 | 72 | 71 | 93 | 96 | 75 | 93 | 99 |  |
| Mupirocin | BHE19_RS06600 | isoleucyl-tRNA synthetase | 100 | 85 | 85 | 85 | 84 | 88 | 81 | 79 | 95 | 97 | 81 | 95 | 99 |  |
| Chloramphenicol | BHE19_RS17100 | hydrophobic/amphiphilic exporter-1, HAE1 family/multidrug efflux pump | 100 | 32 | 32 | 32 | 32 | 84 | 25 | 24 | 93 | 94 | 81 | 95 | 99 |  |
| Elfamycin | BHE19_RS00235 | elongation factor Tu | 100 | 94 | 94 | 94 | 90 | 96 | 96 | 96 | 97 | 99 | 95 | 97 | 100 |  |
| Rifampin | BHE19_RS00195 | DNA-directed RNA polymerase subunit beta | 100 | 95 | 94 | 95 | 95 | 97 | 94 | 94 | 99 | 99 | 96 | 99 | 99 |  |
| Tetracycline | BHE19_RS10130 | 2-polyprenyl-6-methoxyphenol hydroxylase | 100 | 52 | 53 | 53 | 41 | 25 | 0 | 0 | 46 | 84 | 22 | 82 | 98 |  |
| Tetracycline | BHE19_RS18445 | tetracycline resistance monooxygenase | 100 | 47 | 47 | 47 | 40 | 25 | 23 | 25 | 63 | 66 | 0 | 64 | 95 |  |
| Beta-lactamase |  |  |  |  |  |  |  |  |  |  |  |  |  |  |  |  |
|  | BHE19_RS07540 | CubicO group peptidase, beta-lactamase class C family | 100 | 25 | 25 | 25 | 42 | 24 | 35 | 37 | 73 | 77 | 0 | 32 | 99 |  |
|  | BHE19_RS10095 | CubicO group peptidase, beta-lactamase class C family | 100 | 28 | 28 | 28 | 28 | 29 | 26 | 27 | 49 | 91 | 29 | 30 | 98 |  |
|  | BHE19_RS16465 | CubicO group peptidase, beta-lactamase class C family | 100 | 33 | 33 | 33 | 30 | 36 | 32 | 30 | 41 | 80 | 34 | 44 | 96 |  |
|  | BHE19_RS16520 | CubicO group peptidase, beta-lactamase class C family | 100 | 28 | 28 | 28 | 32 | 27 | 30 | 30 | 82 | 48 | 34 | 35 | 99 |  |
|  | BHE19_RS18955 | CubicO group peptidase, beta-lactamase class C family | 100 | 26 | 26 | 26 | 30 | 25 | 23 | 28 | 29 | 43 | 25 | 30 | 98 |  |
|  | BHE19_RS11830 | CubicO group peptidase, beta-lactamase class C family | 100 | 51 | 51 | 51 | 53 | 69 | 51 | 54 | 92 | 89 | 62 | 91 | 100 |  |
|  | BHE19_RS04540 | hypothetical protein | 100 | 73 | 73 | 73 | 29 | 29 | 64 | 62 | 84 | 85 | 66 | 85 | 99 |  |
|  | BHE19_RS08930 | phosphoribosyl 1,2-cyclic phosphate phosphodiesterase | 99 | 73 | 72 | 72 | 74 | 81 | 70 | 68 | 91 | 94 | 67 | 92 | 89 |  |
|  | BHE19_RS12930 | CubicO group peptidase, beta-lactamase class C family | 100 | 75 | 73 | 73 | 67 | 79 | 60 | 57 | 86 | 25 | 69 | 85 | 97 |  |
|  | BHE19_RS19900 | metallo-beta-lactamase class B/metallo-beta-lactamase class B IND | 100 | 0 | 0 | 0 | 0 | 0 | 0 | 0 | 79 | 84 | 0 | 80 | 98 |  |
| Efflux pump conferring antibiotic resistance |  |  |  |  |  |  |  |  |  |  |  |  |  |  |  |  |
|  | BHE19_RS05740 | hydrophobic/amphiphilic exporter-1, HAE1 family | 100 | 39 | 39 | 0 | 40 | 42 | 24 | 25 | 62 | 96 | 31 | 91 | 99 |  |
|  | BHE19_RS12400 | hydrophobic/amphiphilic exporter-1, HAE1 family | 100 | 79 | 79 | 79 | 59 | 61 | 24 | 24 | 95 | 96 | 31 | 96 | 99 |  |
|  | BHE19_RS17385 | hydrophobic/amphiphilic exporter-1, HAE1 family | 100 | 55 | 55 | 55 | 51 | 51 | 23 | 24 | 94 | 93 | 30 | 92 | 99 |  |
|  | BHE19_RS18370 | hydrophobic/amphiphilic exporter-1, HAE1 family | 100 | 48 | 48 | 48 | 61 | 65 | 24 | 25 | 92 | 91 | 31 | 93 | 99 |  |
|  | BHE19_RS18030 | hydrophobic/amphiphilic exporter-1, HAE1 family | 100 | 46 | 46 | 46 | 76 | 91 | 24 | 24 | 93 | 94 | 31 | 94 | 99 |  |
