# Supplementary material for: The Emerging Fish Pathogen Flavobacterium spartansii Isolated from Chinook Salmon: Comparative Genome Analysis and Molecular Manipulation
Source: Front Microbiol. 2017 Nov 30;8:2339. doi: 10.3389/fmicb.2017.02339 (PMC5714932; doi:10.3389/fmicb.2017.02339)
Supplement: Supplementary file 2 [file DataSheet1.docx]

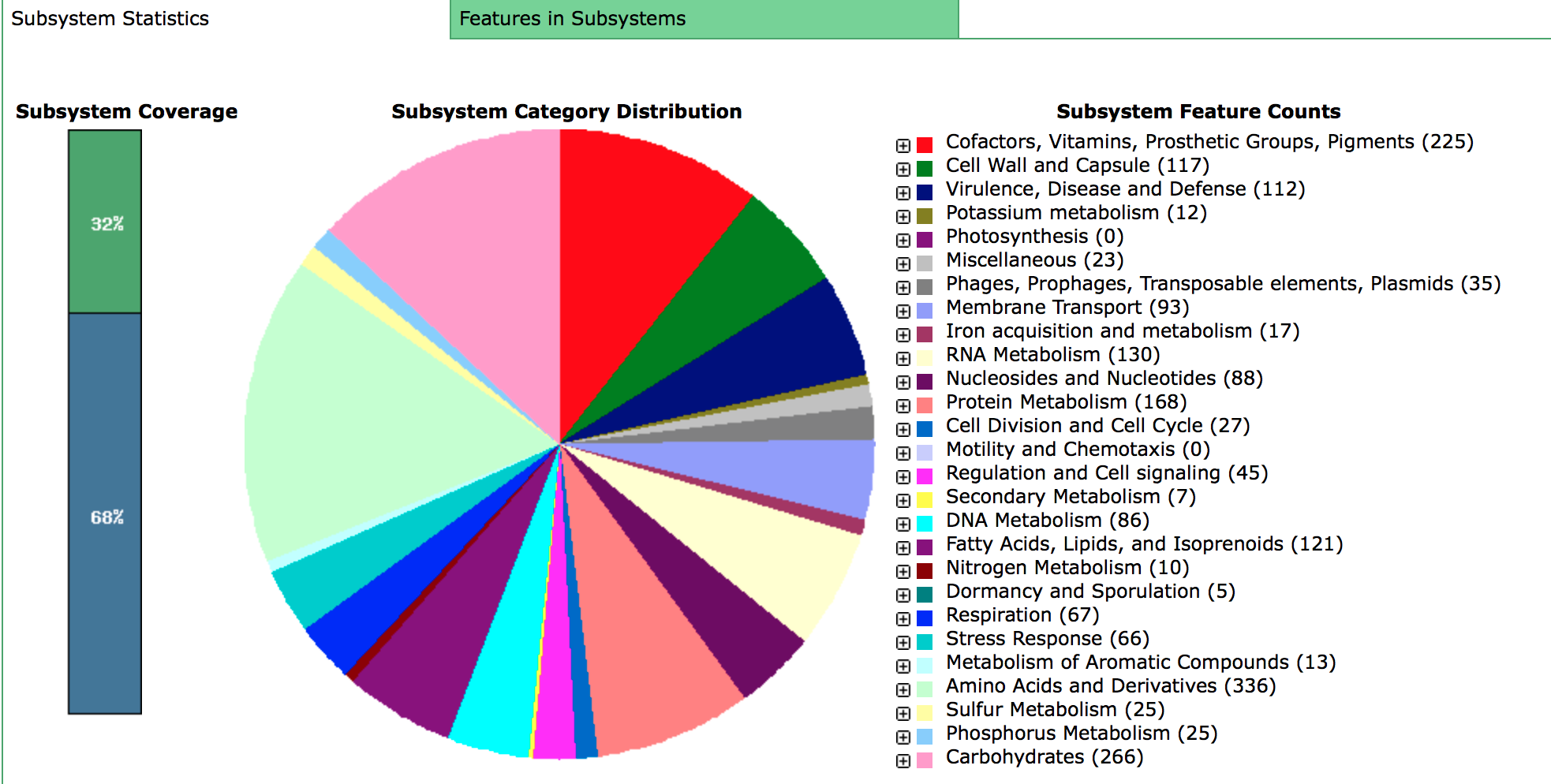


**Figure S1. The Subsystem distribution in different categories of *F. spartansii* T16^T^.** Subsystem coverage shows the total genes in the subsystems (32% in subsystems and 68% not in subsystems). Each part of the pie graph indicates different functions and proportions of genes. The numbers in parentheses show the counts of genes with specific functions.
